# Estrogenic vascular effects are diminished by chronological aging

**DOI:** 10.1038/s41598-017-12153-5

**Published:** 2017-09-22

**Authors:** Christopher J. Nicholson, Michèle Sweeney, Stephen C. Robson, Michael J. Taggart

**Affiliations:** 1 Institute of Cellular Medicine, Newcastle University, Newcastle Upon Tyne, UK; 2 Cardiovascular Research Centre, Institute of Genetic Medicine, Newcastle University, Newcastle Upon Tyne, UK; 3 School of Biomedical Sciences, Newcastle University, Newcastle Upon Tyne, UK

## Abstract

The beneficial role of estrogen in the vascular system may be due, in part, through reduction of peripheral vascular resistance. The use of estrogen therapy to prevent cardiovascular disease in post-menopausal women remains contentious. This study investigated the influence of aging and the menopause on the acute vasodilatory effects of estrogen using *ex vivo* human and murine resistance arteries. Vessels were obtained from young (2.9 ± 0.1 months) and aged (24.2 ± 0.1 and 28.9 ± 0.3 months) female mice and pre- (42.3 ± 0.5 years) and post-menopausal (61.9 ± 0.9 years) women. Aging was associated with profound structural alterations of murine uterine arteries, including the occurrence of outward hypertrophic remodeling and increased stiffness. Endothelial and smooth muscle function were diminished in uterine (and tail) arteries from aged mice and post-menopausal women. The acute vasodilatory effects of 17β-estradiol (non-specific estrogen receptor (ER) agonist), PPT (ERα-specific agonist) and DPN (ERβ-specific agonist) on resistance arteries were attenuated by aging and the menopause. However, the impairment of estrogenic relaxation was evident after the occurrence of age-related endothelial dysfunction and diminished distensibility. The data indicate, therefore, that chronological resistance arterial aging is a prominent factor leading to weakened vasodilatory action of estrogenic compounds.

## Introduction

Cardiovascular diseases (CVDs) are the leading cause of death worldwide^1^. Age is the biggest determinant of an individual’s cardiovascular health and since the number of older people is growing substantially, there is a need to develop a greater understanding of the influence of age on the cardiovascular system^2^. There are also marked differences in the prevalence of CVD in men and women; prior to 50 years of age, the average age of menopause onset, the risk is considerably lower in women but thereafter the incidence is similar^3,4^. Post-menopausal deprivation of female sex hormones, primarily estrogen, may be related to the increased CVD risk in aging women^5^.

Estrogen binds to two receptors, ERα and ERβ, which can then initiate ligand-activated transcription factor signaling^6^. However, estrogen can also induce acute arterial vasodilation separate from genomic-directed actions^7–13^. It has been proposed that estrogen activates endothelial nitric oxide synthase (and thus NO production), endothelial prostacyclin and endothelium-derived hyperpolarizing (EDH) factor, which act on adjacent smooth muscle cells to induce relaxation^7–13^. Indeed, it has been suggested that the contribution of EDH is greater in females due to the presence of estrogen^14–16^. Further to acting through the classical ERs, estrogen has been demonstrated to act through the unrelated G-protein-coupled estrogen receptor (GPER1) to induce vasodilation^17–21^. In large arteries, the effects of estrogen are considered to protect against inflammatory-related disorders such as atherosclerosis, whereas in the resistance vasculature, the acute vasodilatory actions may lower peripheral resistance and protect against hypertension^22^.

Consistent with the proposed cardiovascular protective role of estrogen, early observational studies, such as the Nurses’ Health Study, suggested estrogen therapy might reduce the risk of CVD in post-menopausal women^23^. However, randomized clinical trials largely failed to show any cardiovascular benefits of menopausal hormone therapy (MHT) in post-menopausal women, with guidelines concluding that MHT should not be used for CVD prevention^24–26^. However, subsequent re-analysis of the Women’s Health Initiative data revealed a trend for a lower risk of coronary heart disease in hormone-treated women from the youngest age group. This suggested the possibility of a ‘timing’ hypothesis wherein MHT initiated within 10 years of the menopause could be beneficial to CVD health^27–29^. Indeed, a recent study suggested there to be some beneficial cardiovascular effects of estradiol treatment in early (<6 years) menopausal compared to late (≥ 10 years) menopausal women^30^. Recent evidence has also highlighted the role for aging per se, independent of menopausal status, in female cardiovascular risk^31,32^. Thus, it remains an important objective to elucidate the relative contributions of aging and the menopause, and the influence of estrogenic compounds, on vascular function.

Therefore, in the present study we have sought to determine the contributions of age or the menopause on arterial function and estrogenic responsiveness *in vitro*. To do so we employed two experimental models of arterial vascular function; uterine and tail arteries from young (3 months) and old (24 and 29 months) mice, to determine the influence of age, and uterine arteries from non-pregnant women ranging in age from 32–75 years, to assess the influence of menopause and/or age.

## Results

### Aging negatively regulates murine uterine arterial function and passive structural characteristics

Prior to testing the influence of age on estrogenic responsiveness of murine resistance arteries, we sought to determine the role of aging on vasoactive and structural properties of these vessels. We utilized mice from the C57BL background (C57BL/Ircfa) from 3 separate age groups (Table 1). These mice remain generally healthy into old age with no strain-specific pathological conditions^33–36^. We chose to study two different aged groups (24 and 29 months) in order to assess the influence of advanced age on resistance artery structure and function, since it was not often possible to obtain samples from very old women. This 5-month difference is equivalent of human aging from 69 to 80 years of age^37^ and, therefore, from an observational standpoint one would expect an acceleration of age-related dysfunction. Indeed, percent survival of this mouse strain decreases considerably in this timespan^36^.

**Table 1:** Mouse details.

Characteristics	3 months (n = 26)	24 months (n = 10)	29 months (n = 5)
**Age** (**months**)	2.91 ± 0.09	24.18 ± 0.14*	28.86 ± 0.28*^†^
**Weight** (**g**)	22.06 ± 0.08	26.83 ± 0.44*	30.48 ± 1.48*^†^
**Uterine weight** (**g**)	0.46 ± 0.02	0.67 ± 0.05*	0.34 ± 0.06^†^
**Heart weight** (**g**)	0.16 ± 0.01	0.16 ± 0.00	0.16 ± 0.01
**Estrous stage:**			
Pro-estrus	9	—	—
Estrus	6	—	—
Metestrus	4	—	—
Diestrus	7	10	5
**Resting diameter** (**μm**)**:**			
Uterine arteries	164 ± 4.5	188.5 ± 4.0*	211.0 ± 5.0*
Tail arteries	251.7 ± 6.3	227.0 ± 5.5*	265.9 ± 9.7^†^

All values, apart from estrous stage, are presented as mean ± SEM. *P < 0.05 from young mice (ordinary one-way ANOVA). ^†^P < 0.05 from 24-month-old mice (ordinary one-way ANOVA).

**Figure 1:**
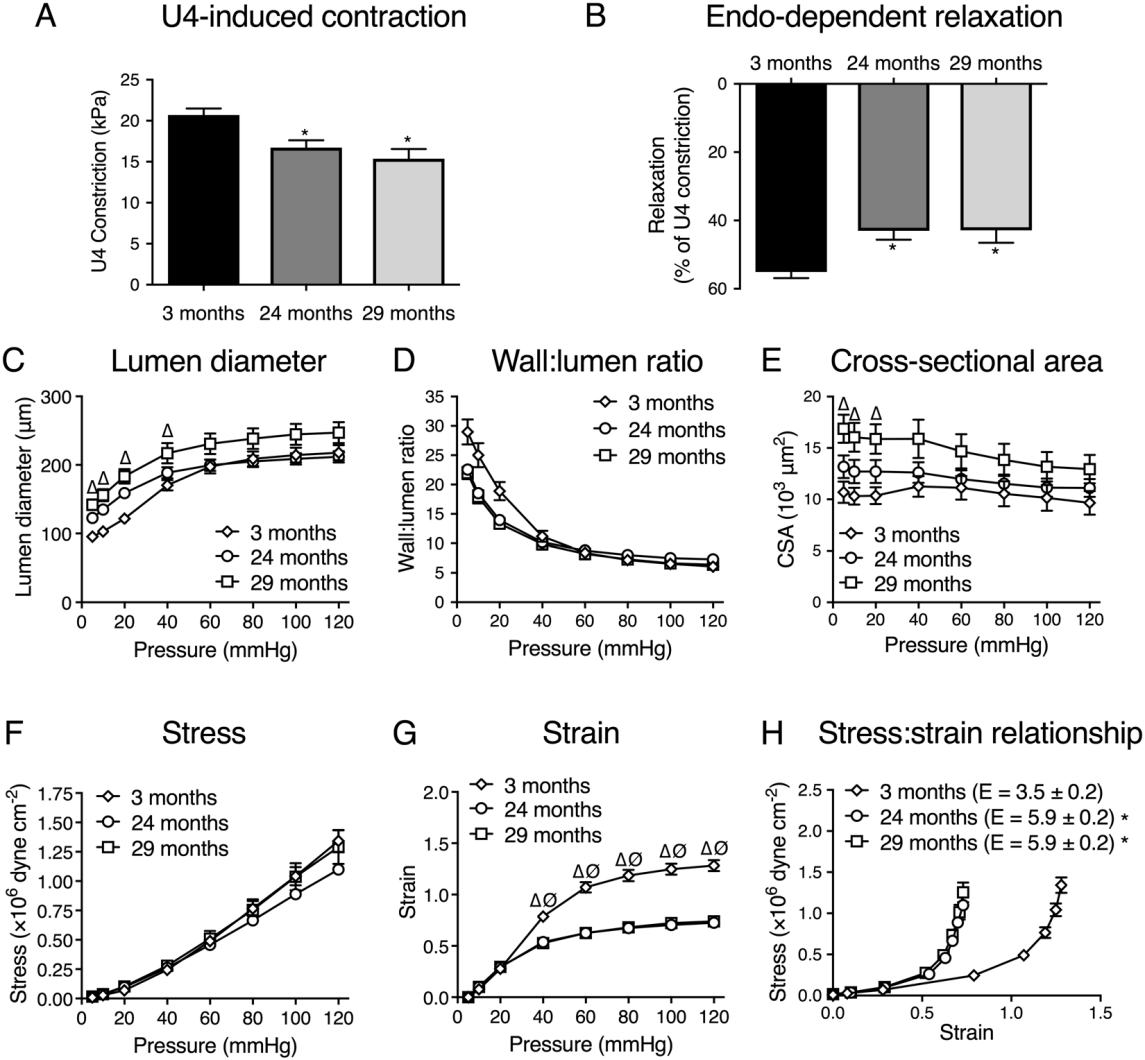
Aging alters the vasoactive and passive structural characteristics of murine uterine arteries. For the vasoactive experiments, uterine arteries from 3- (n = 26), 24- (n = 10), and 29-month-old (n = 5) mice were mounted on a wire myograph and exposed to the thromboxane agonist U4 (10^−6^M, **A**). Following steady-state constriction, endothelium-dependent relaxation was assessed by the addition of acetylcholine (10^−5^M, **B**). *P < 0.05 from young mice (ordinary one-way ANOVA). For the assessment of passive structural characteristics, separate uterine arteries from 3- (n = 5), 24- (n = 7), and 29-month-old (n = 4) mice were mounted on a pressure myograph and measurements of (**C**) vessel diameter and wall thickness were used to calculate (**D**) wall: lumen ratio, (**E**) cross-sectional area (CSA), (**F**) vessel stress and (**G**) strain at each pressure step (0–120 mmHg) in calcium-free conditions. P < 0.05 from 24-month-old mice (Ø) or 29-month-old mice (∆) (repeated measures two-way ANOVA). Vessel stress was plotted against vessel strain to assess the stress: strain relationship, which is used to ascertain arterial distensibility (**H**). E = Elastic modulus. *P < 0.05 from young mice (unpaired t-test). Data are presented as mean ± SEM.

Vasoactive responses to the thromboxane agonist U46619 (U4) and the endothelium-dependent vasodilator acetylcholine (ACh), and the passive structural responses to increasing intravascular pressure (illustrated in Fig. 1) were assessed. Both U4-induced constriction (maximum contractions for 3-month-old: 20.7 ± 0.8 kPa, 24-month-old: 16.7 ± 0.9 kPa and 29-month-old: 15.4 ± 1.2 kPa, Fig. 1A) and endothelium-dependent vasodilation (maximum relaxations for 3-month-old: 55.1 ± 1.8%, 24-month-old: 42.1 ± 2.4% and 29-month-old: 42.9 ± 3.6%, Fig. 1B) were attenuated in aged mice.

The effect of aging on passive structural characteristics was more complex. There was no difference in the passive lumen diameter of uterine arteries from 3- and 24-month-old mice. However, further aging to 29 months increased the passive diameter of uterine arteries (Fig. 1C), which was associated with an increase in cross-sectional area (CSA) (Fig. 1E). There was also a slight, but non-statistically significant, decrease in the wall-to-lumen ratio (Fig. 1D). Increases in passive diameter and CSA are consistent with outward hypertrophic remodeling. Aging was also associated with a decrease in the strain: pressure relationship (Fig. 1G) with no change in the stress: pressure relationship (Fig. 1F). Consequently, there was an increased elastic modulus in arteries from aged mice (Fig. 1H), indicating a reduced arterial distensibility (i.e. increased stiffness). This is supported by a leftward shift in the stress: strain curve in uterine arteries from aged mice (Fig. 1H).

### Estrogenic responsiveness of murine uterine arteries is impaired in the oldest mice, after the occurrence of endothelial dysfunction and increased stiffness

**Figure 2﻿:**
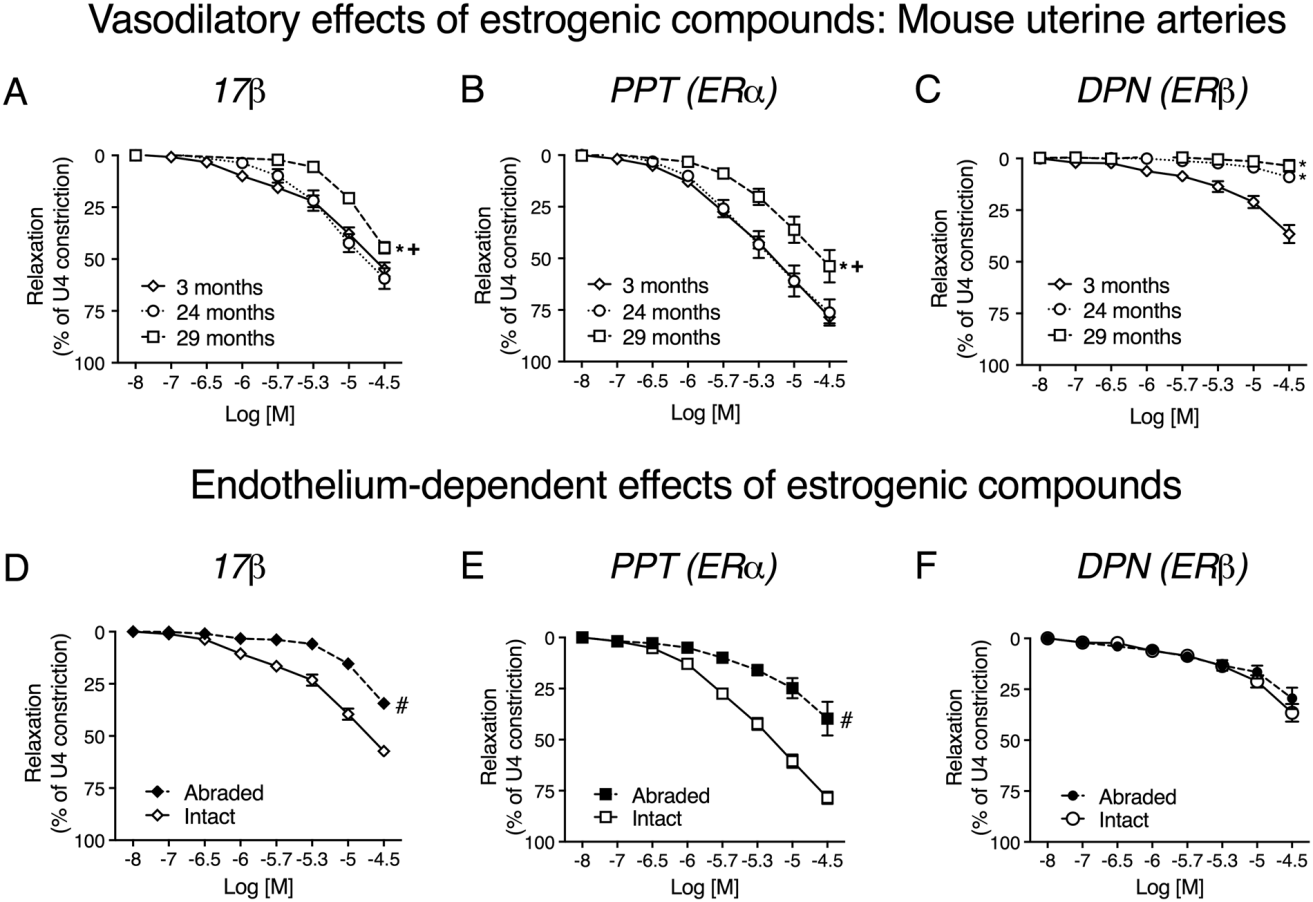
﻿The acute responses of murine uterine arteries to estrogenic compounds are attenuated by aging. Acute relaxations to 17β (**A**), PPT (**B**) and DPN (**C**) were compared in uterine arteries from 3- (n = 26), 24- (n = 9) and 29-month-old (n = 5) female mice. P < 0.05 from young mice (*) or 24-month-old mice (+) (repeated measures two-way ANOVA). In a separate set of experiments, pre-constricted (U4, 10^−6^M) endothelium-intact (solid lines and open symbols, n = 26) and endothelium-abraded (dashed lines and closed symbols, n = 7) uterine arteries from young mice were evaluated for their responses to incremental doses (10^−8^M−10^−4.5^M) of 17β-estradiol (**D**), PPT (**E**) or DPN (**F**). # P < 0.05 vs. intact arteries (repeated measures two-way ANOVA). Data are presented as mean ± SEM.

We examined the effect of aging on the vasodilatory responses of resistance arteries to estrogenic compounds. Figure 2 demonstrates the vasorelaxant effects of 17β-estradiol (17β), PPT (ERα) and DPN (ERβ) on pre-constricted uterine arteries from 3-, 24- and 29-month-old mice. Aging was associated with attenuation of the acute vasodilatory properties of all three estrogenic compounds but the effects were agonist-specific. The acute vasodilatory effects of 17β and PPT were not different between 3 and 24 months but were reduced when comparing 3- and 29-month-old mice. In addition, the effects of both 17β-estradiol and PPT were greater in arteries from 24-month-old when compared to 29-month-old mice. Therefore, impaired relaxations to estrogenic compounds occurred after altered functional and elastic arterial properties of these vessels were demonstrable (see Fig. 1).

Comparing Fig. 2A, B and C, it appears there are differences in the effects of the estrogenic compounds. Indeed, the acute vasodilatory effects of PPT were greater than those of 17β, which elicited a more pronounced response than DPN (repeated measures two-way ANOVA).

Having determined that endothelial dysfunction was associated with aging in murine resistance arteries, we hypothesized that the reduced vasorelaxant effects of estrogenic compounds may be attributable, in part, to altered endothelial-mediated relaxation. We therefore examined the relaxatory effects of each estrogenic compound in endothelium-intact and endothelium -abraded uterine vessels from young mice (demonstrated in Fig. 2D–F). The acute vasodilatory effects of 17β (intact: 17.2 ± 3.7% to 50.8 ± 3.9%; abraded: 15.4 ± 2.7% to 34.4 ± 4.8%, Fig. 2D) and PPT (intact: 16.7 ± 1.4% to 87.5 ± 4.0%; abraded: 24.8 ± 4.9% to 39.7 ± 8.2%, Fig. 2E) were blunted following endothelial abrasion. The acute vasodilatory effect of DPN did not differ between groups, possibly due to the minimal responses observed to this agonist (intact: 11.4 ± 3.0% to 36.1 ± 4.6%; abraded: 16.5 ± 3.1% to 29.4 ± 5.2%, Fig. 2F).

### Estrogenic vasodilation is also impaired in non-uterine arteries from aged mice

**Figure 3﻿:**
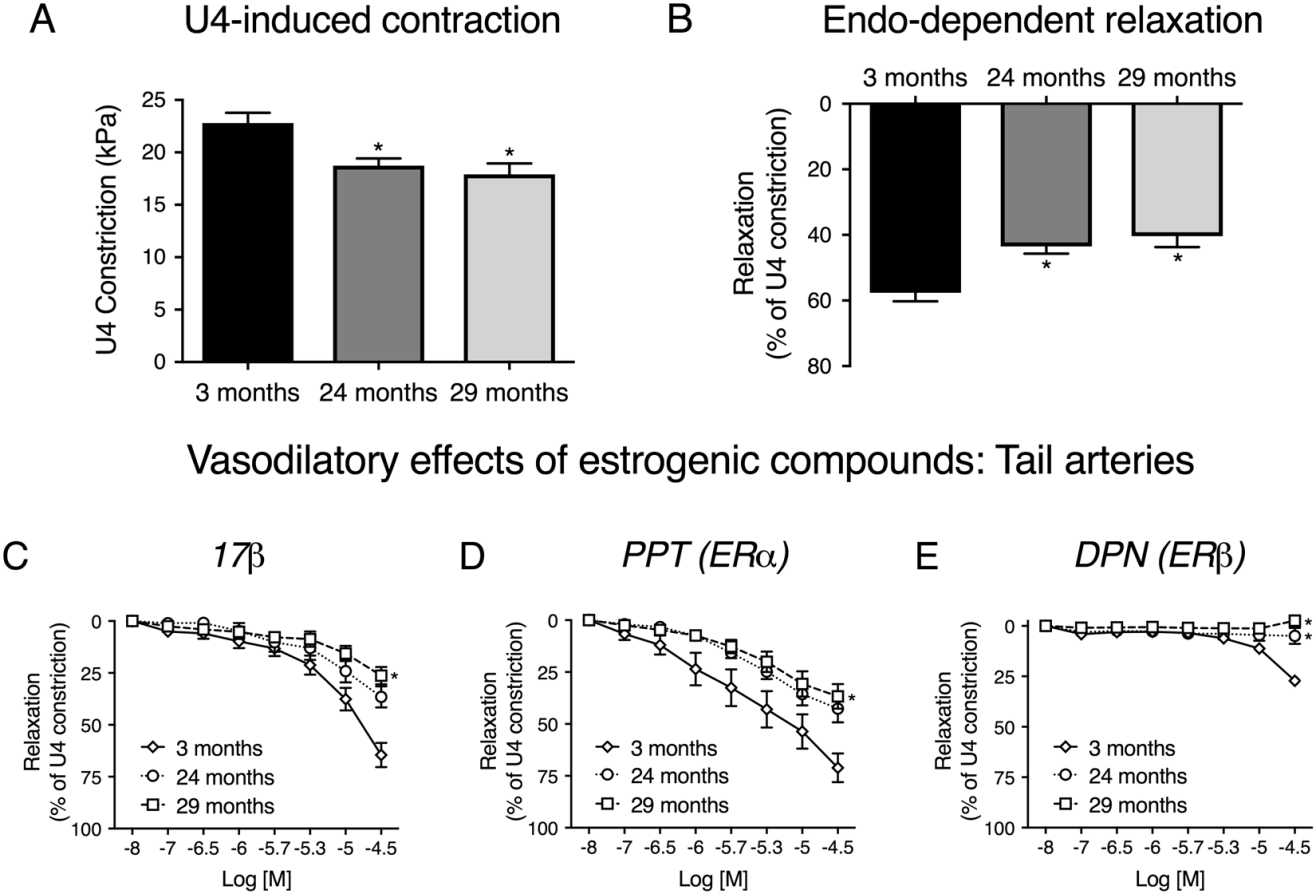
﻿The acute responses of murine tail arteries to estrogenic compounds are impaired by aging. Tail arteries from 3- (n = 12), 24- (n = 10) and 29-month-old (n = 5) mice were mounted on a wire myograph and exposed to the thromboxane agonist U4 (10^−6^M, **A**). Following steady-state constriction, endothelium-dependent relaxation was assessed by the addition of acetylcholine (10^−5^M, **B**). *P    < 0.05 from young mice (unpaired t-test). Acu﻿te relaxations to 17β (**C**), PPT (**D**) and DPN (**E**) were compared in tail arteries from 3- (n = 8), 24- (n = 9) and 29-month-old (n = 5) female mice. *P < 0.05 from young mice (repeated measures two-way ANOVA). Data are presented as mean ± SEM.

In order to determine if the influence of aging on estrogenic vasodilation in uterine arteries was observable in other resistance vessels, we repeated studies on tail arteries from female mice. This is important, since there is considerable vascular bed-heterogeneity in the effects of estrogenic compounds^9,22,38^. Figure 3 illustrates tail arterial responses to U4, ACh, 17β, PPT and DPN from 3-, 24- and 29-month-old mice. Similar to uterine arteries, both U4-induced constriction (maximum contractions for 3-month-old: 22.8 ± 1.0 kPa, 24-month-old: 18.7 ± 0.7 kPa and 29-month-old: 17.9 ± 1.0 kPa, Fig. 3A) and endothelium-dependent vasodilation (maximum relaxations for 3-month-old: 57.7 ± 2.6%, 24-month-old: 43.5 ± 2.2% and 29-month-old: 40.4 ± 3.4%, Fig. 3B) were attenuated in aged mice. The acute relaxatory effects of 17β (Fig. 3C) and PPT (Fig. 3D) were greater than those of DPN (Fig. 3E) (repeated measures two-way ANOVA). Further, the acute estrogenic effects of 17β and PPT were attenuated with advanced aging to 29 months, similar to uterine arteries. However, there was a trend for a decrease in the responses of tail arteries from 24-month-old mice.

### Human arterial function and estrogenic responsiveness is weakened in post-menopausal women

**Figure 4﻿:**
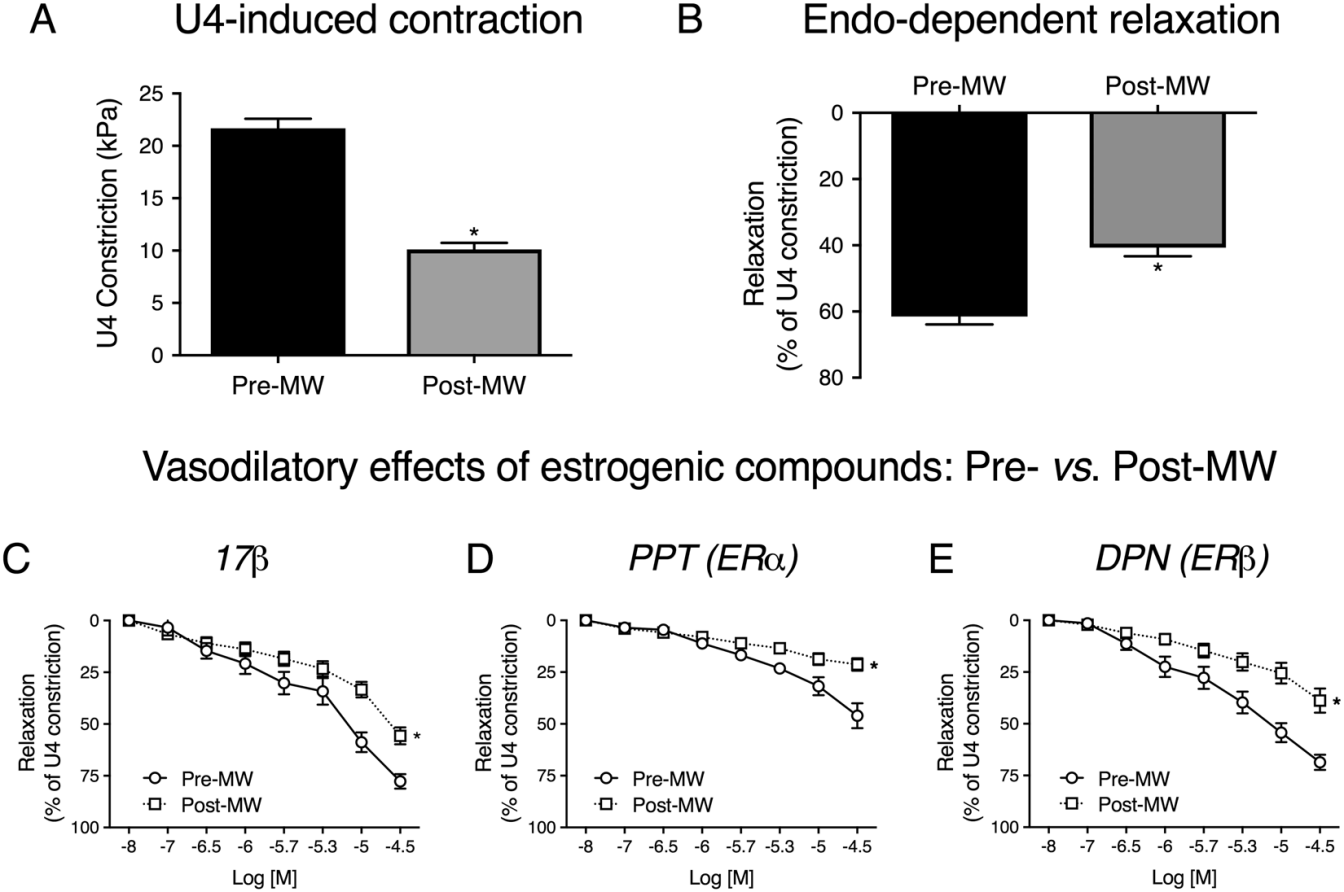
Vasoactive properties and estrogenic responsiveness of human uterine arteries are attenuated in post-menopausal women. Uterine arteries were mounted on a wire myograph and exposed to the thromboxane agonist U4 (10^−6^M, **A**). Following steady-sta﻿te constriction, endothelium-dependent relaxation was assessed by the addition of bradykinin (10^−5^M, **B**). Contractility and endothelial-dependent relaxations were compared in arteries from pre- (n = 26) and post-menopausal (n = 16) women. *P < 0.05 from pre-menopausal women (unpaired t-test). Acute relaxations to 17β (**C**), PPT (**D**) and DPN (**E**) were compared in uterine arteries from pre- (n = 20) and post-menopausal women (n = 13). *P < 0.05 from pre-menopausal women (repeated measures two-way ANOVA). Pre-MW = pre-menopausal women. Post-MW = post-menopausal women. Data are presented as mean ± SEM.

To determine whether the menopause influences vasoreactivity of resistance arteries from women, we studied the responses of uterine arteries from pre- and post-menopausal women from the ages of 32 to 75 years (Fig. 4). The menopause was associated with impaired U4-induced contractility (maximum contractions for pre-menopausal: 21.7 ± 0.9 kPa and post-menopausal: 10.1 ± 0.6 kPa, Fig. 4A) and endothelium-dependent relaxations (maximum relaxations for pre-menopausal: 61.5 ± 2.4% and post-menopausal: 40.7 ± 2.6%, Fig. 4B) to bradykinin.

In contrast to murine resistance arteries, the relaxations induced by 17β-oestradiol (Fig. 4C) and DPN (Fig. 4E) were greater than those induced by PPT (Fig. 4D) in arteries from pre-menopausal women (repeated measures two-way ANOVA). Importantly, we found that the acute vasodilatory effects of all three compounds were attenuated in uterine arteries from post-menopausal women.

### Human uterine arterial function is impaired with increasing age

**Figure 5:**
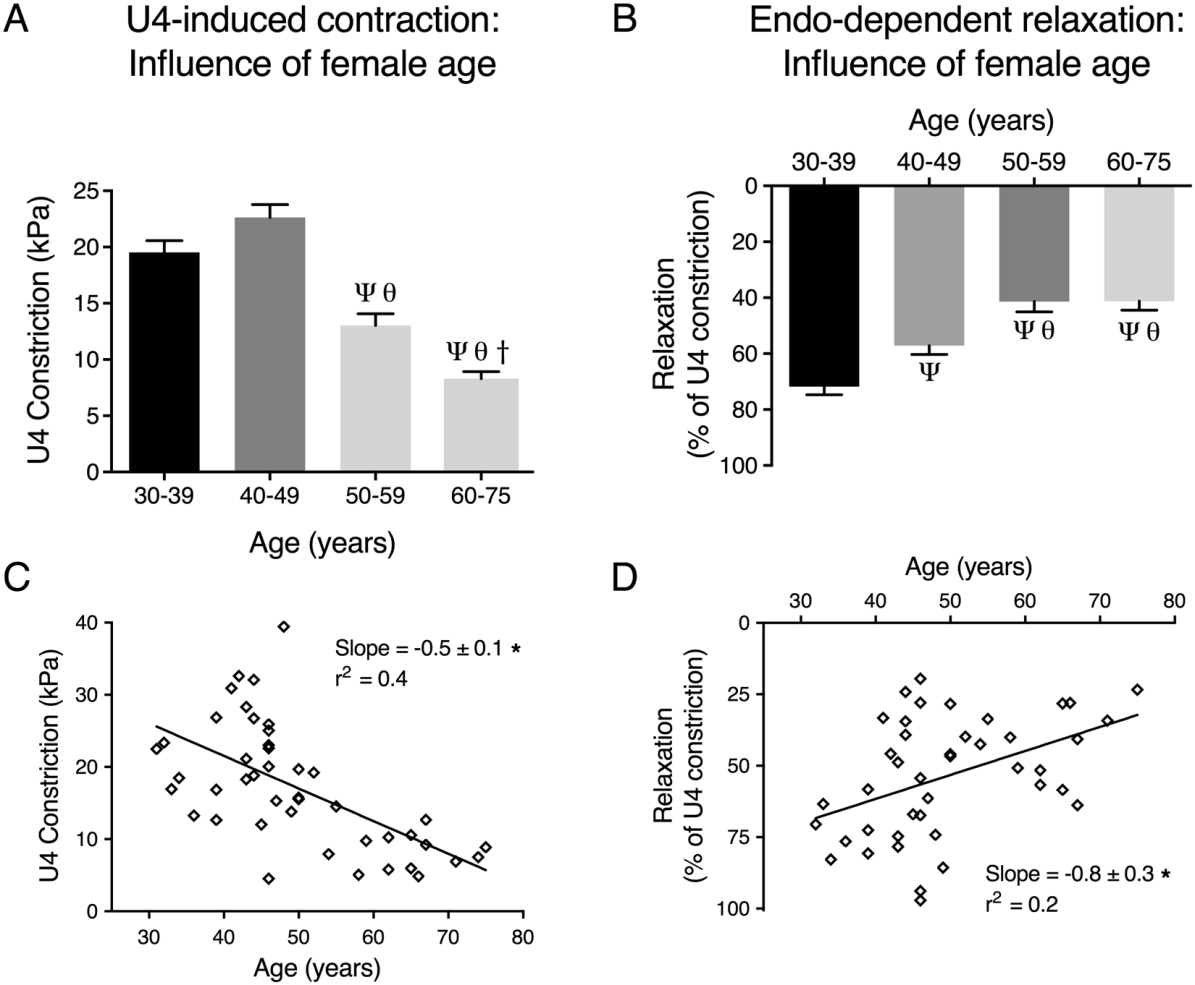
Human uterine arterial function is impaired with increasing age. Maximal U4-induced contractions (**A**) and endothelial-dependent relaxations (**B**) were comp﻿ared in 4 age groups (30-39, 40-49, 50-59 and 60-75 years of age). P < 0.05 from; 30-39 (Ψ), 40-49 (θ), 50-59 (†) (ordinary one-way ANOVA). Scatter plots show (**C**) the mean maximal U4-induced constriction and (**D**) endothelial-dependent relaxation of uterine arteries from each individual patient plotted against age. *P < 0.05 from zero slope (F test). The slope and, r^2^ values are presented in each XY scatter graph.

The data presented in Fig. 4 were re-plotted to further examine the effect of aging on vasoreactivity of uterine resistance arteries from women. Figure 5 demonstrates U4-induced contractions (A) and endothelial-dependent relaxations (B) in women grouped into 4 age groups (years): 30–39 (n = 7), 40–49 (n = 18), 50–59 (n = 8) and 60–75 (n = 9). Maximal U4-induced contractions were similar in women under the age of 50, but were diminished in women over 50 (maximal contractions for 30–39: 19.5 ± 1.0 kPa, 40–49: 22.6 ± 1.1 kPa, 50–59: 13.0 ± 1.0 kPa and 60–75: 8.3 ± 0.6 kPa). Interestingly, maximal endothelial-dependent relaxations were strongest in the youngest group of women and diminished in all others (maximal relaxations for 30–39: 71.8 ± 2.9%, 40–49: 57.1 ± 3.2%, 50–59: 41.4 ± 3.6% and 60–75: 41.2 ± 3.3%). Increasing age negatively influenced both smooth muscle and endothelial function (Fig. 5C and D).

### Age-related endothelial dysfunction of human uterine arteries precedes changes in estrogenic responsivness

**Figure 6﻿﻿:**
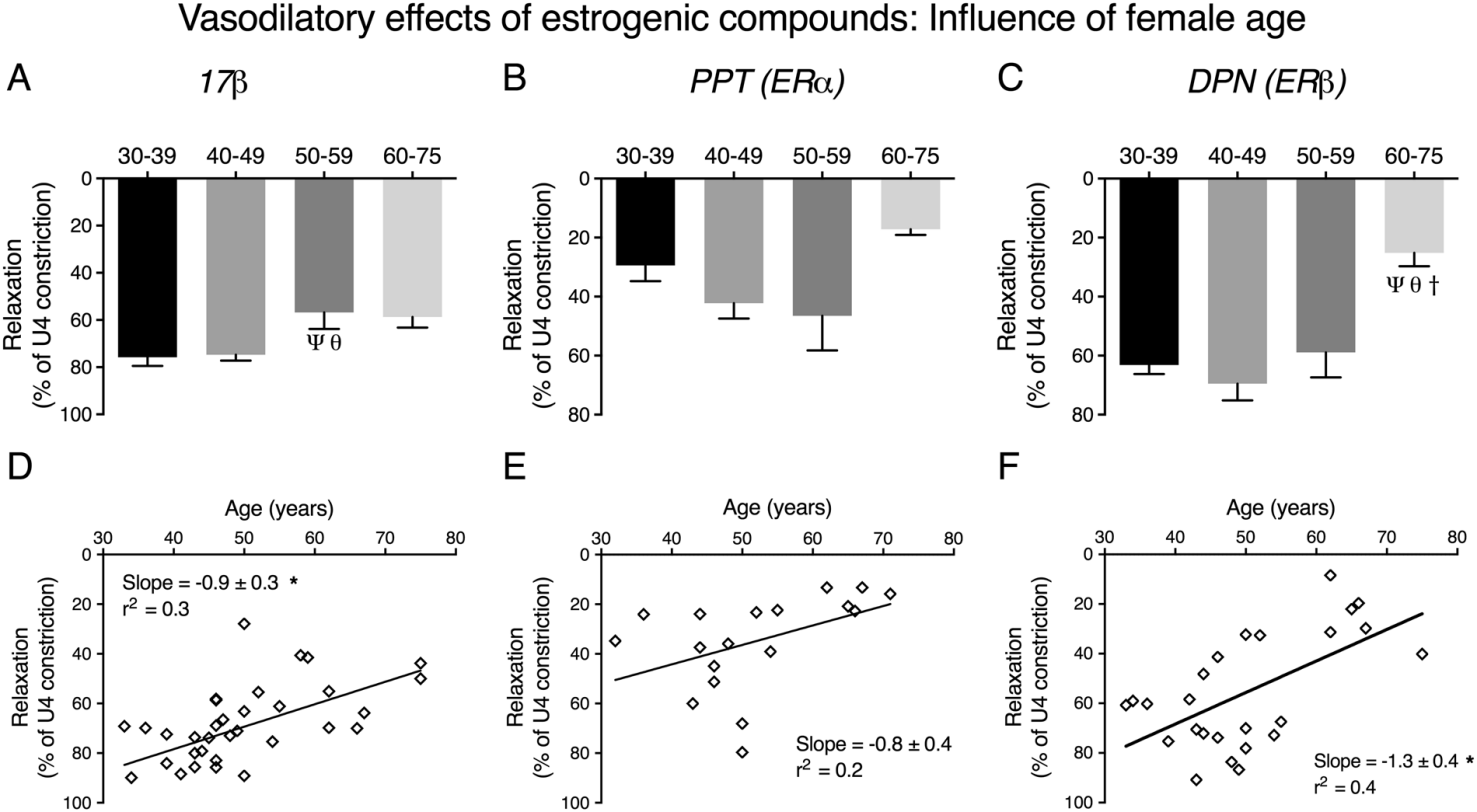
Aging negatively regulates the acute vasodilatory effects of estrogenic compounds in women. Maximal acute relaxations to 17β (**A**), PPT (**B**) and DPN (**C**) were compared in 4 age groups (30-39, 40-49, 50-59 and 60-75 years of age). P < 0.05 from; 30-39 years old (Ψ), 40-49 years old (θ), 50-59 years old (†) (ordinary one-way ANOVA). Scatter plots show the maximal relaxations from each patient plotted against increasing age. *P < 0.05 from zero slope (F test). The slope and r^2^ values are presented in each XY scatter graph.

Note: due to the variable nature of myometrial samples provided, only a small number of vessels could be isolated from some biopsies. Therefore, in some experiments it was not possible to assess the effects of all three compounds. This is why there is a greater number of data points for 17β, which we included in every experiment.

The effects of aging on the acute responses of human uterine arteries to estrogenic compounds were similarly assessed in female age groups (years): 30–39 (n = 5), 40–49 (n = 15), 50–59 (n = 8) and 60–75 (n = 6) (using data re-analyzed from Fig. 4). As shown in Fig. 6A–C, the maximal relaxatory effects of 17β (maximal relaxations for 30–39: 77.1 ± 4.2%, 40–49: 78.2 ± 4.3%, 50–59: 56.8 ± 7.1% and 60–75: 58.8 ± 4.4%) and DPN (maximal relaxations for 30–39: 63.8 ± 3.8%, 40–49: 69.5 ± 5.7%, 50–59: 58.9 ± 8.5% and 60–75: 25.2 ± 4.5%) were negatively influenced by increasing age. Interestingly, whereas the vasodilatory effect of 17β was impaired in women over the age of 50, a weakening of the effects of the ER-specific agonists appeared in women over the age of 60. Similar to murine arteries, the weakened responses of human arteries to these agonists were only apparent after the occurrence of endothelial dysfunction. In addition, there was a negative trend between vasodilatory responses of human uterine arteries to estrogenic compounds and aging (Fig. 6D and F).

## Discussion

The acute vasodilatory effects of estrogen have previously been established but there is little information about how these arterial responses are influenced by the menopause and advancing age. This study investigated the influence of chronological age and the menopause on the acute vasodilatory effects of 17β and ER-specific agonists. Our data demonstrated that both aging and the menopause were associated with reduced vasodilatory effects of estrogenic compounds in murine and human uterine arteries, respectively. Interestingly, however, aging altered endothelial function prior to estrogenic responsiveness in human resistance arteries. Similarly, in mice, aging was associated with reduced resistance arterial compliance, occurring before the altered effects of estrogenic compounds became apparent. We therefore propose that age-related changes in arterial endothelial function and/or stiffness are prominent factors, and not just menopausal status per se, leading to impaired estrogenic actions in arteries from post-menopausal women.

We report that the acute vasodilatory effects of estrogenic compounds, 17β-estradiol and ER agonists, are impaired in resistance arteries from post-menopausal women. An important consideration is what contribution aging per se may make to these findings. Indeed, sub-group analysis of the data suggested that chronological aging negatively influenced the estrogenic responsiveness of human uterine arteries. In particular, endothelial-dependent relaxations were greater in women aged 30–39 than those aged 40–49. Therefore, endothelial dysfunction of human resistance arteries occurred prior to the defect in estrogenic responsiveness, and also the menopause. Due to the small study numbers in humans, we were not able to fully dissociate the influence of age and the menopause on human uterine vascular function. Therefore, we also examined the influence of chronological age on vascular responsiveness in female mice. The data, from two different vascular beds, also demonstrated age-related impairment of acute responses to 17β-estradiol, PPT and DPN. Similar reductions in vascular relaxation to 17β-estradiol have been reported for rat vessels^21,39^. Of note, the most pronounced effects of aging on 17β- and PPT-induced relaxations of murine uterine arteries were observed between 24 and 29 months rather than between 3 and 24 months. This impairment of estrogenic responsiveness occurred mostly after the endothelial dysfunction and altered distensibility, each evident at 24 months, were established. Therefore, in alliance with the literature^21,31,32,39,40^, our data supports the concept that the vascular aging phenotype is a continuum of many facets and that the marked structural and functional changes evident at 24 months impose extra strain that contributes to detrimental effects on estrogenic responsiveness.

It is important to note that the current study demonstrated the maximum vasodilatory effects of estrogenic compounds at micromolar concentrations. Similar ranges of concentrations have been used in previous studies^7,10,11,38,39,41^. It is also important to note that, since estrogen is a lipophilic molecule, the concentrations of oestradiol are possibly greater at the cellular site of action than those measured in the circulation.

We also demonstrated that outward hypertrophic remodeling of uterine arteries was evident in the oldest group of mice, suggesting changes to wall structure may be a response to worsened arterial compliance of resistance vessels. It has been suggested that hypertrophic remodelling is associated with a reduced myogenic response of resistance arteries^42^. Previous studies investigating the effect of age on contractile properties of resistance arteries have been conflicting, with the over-arching hypothesis that the effects are species-, tissue- and even agonist-specific^40,43–47^. However, a diminution in response to U46619 was a consistent feature of our data in resistance vessels from humans and mice.

The structural properties of human uterine arteries were not measured in the current study. Therefore, it is not known whether human uterine resistance arteries displayed outward hypertrophic remodelling with age. However, the functional studies indicate similar effects of age, and estrogen responsiveness, in human and mouse uterine arteries. As such it is tempting to suggest that the human arteries may evince similar structural changes to those measured in the mouse arteries from two separate vascular beds. However, others have shown that small arteries from gluteal biopsies display progressive eutrophic remodeling with age^48,49^.

Total peripheral resistance is an important determinant of blood pressure. The incidence of hypertension is increased with aging and further contributes to the risk of developing cardiovascular disease^50,51^. In addition, in mixed gender human studies using gluteal biopsies, eutrophic inward remodeling has been suggested to accompany chronological aging and essential hypertension^48^. However, none of the female human subjects in this study were reported to be hypertensive. Further, although we were unable to monitor blood pressure in our mouse studies, it is unlikely that hypertension was a contributory factor to the alterations in structure/function since we observed outward hypertrophic remodeling.

Previous studies have attributed age-related blunting of estrogenic vasodilation to a reduction in the expression of GPER1 and ERs^21,39,52,53^. In the current study, we found no difference in ER expression levels with age in human resistance arteries (supplementary Figure 2). In addition, we found that the GPER1 agonist G1 had no effect in human uterine arteries (supplementary Figure 1), supporting a previous study using uterine arteries from pregnant women^9^. Further, the data enables the speculation that estrogenic relaxation is mediated by predominantly ERα in mouse resistance arteries and ERβ in human uterine arteries. This contributes to the notion that similar estrogenic vascular responsiveness may be mediated in ER-specific manners^38^.

Alterations in arterial stiffness and endothelial dysfunction are often observed in unison and the former is a strong independent indicator for subsequent cardiovascular risk^54^. Endothelial dysfunction with aging has been associated with impaired nitric oxide (NO)- and EDH-mediated relaxation, each of which is a potential target of modulation by estrogenic compounds^39,55,56^. Either way, endothelial dysfunction, or deliberate removal of endothelial-dependent relaxation, resulted in a blunting of estrogenic responsiveness.

There is currently much debate as to the role of the menopause, and altered estrogen bioavailability, in determining risks for cardiovascular health and attenuation in estrogen-responsiveness of the resistance vasculature. The findings reported herein, have implications for the interpretation of clinical studies examining cardiovascular outcomes in aging females. Certainly, estrogen withdrawal has been reported to influence vascular gene expression, inflammation, oxidative stress, structure and function^8,22,57–64^. There is also some evidence that estrogen therapy administered to women soon after the menopause may be of more benefit than if treatment is delayed^27,29,65,66^. Recently, two clinical trials, the ‘Kronos Early Estrogen Prevention Study’ (KEEPS) and ‘Early versus Late Intervention Trial with Oestradiol’ (ELITE) trials, have been designed to assess if the length of time since menopause alters the effectiveness of estrogen therapy in post-menopausal women. Early data from the KEEPS trial has shown no significant improvement on cardiovascular mortality, endothelial function or carotid artery compliance of estrogen therapy in young post-menopausal women^67–69^. However, clinical data from the ELITE trial demonstrated that oestradiol treatment had a beneficial effect on carotid artery intima-medial thickness in recently menopausal women (<6 years) compared with distantly menopausal women (≥10 years), yet outcome measures of coronary atherosclerosis risk were unaffected^30^.

These studies necessitate a continued appraisal of the likely impact of changes in cardiovascular function related to age in women. For example, although long-term risks of adverse cardiovascular outcomes may be similar for post-menopausal women and males there is increasing evidence of key gender differences. These include: (i) first cardiovascular events occurring at older ages in females and the risk of coronary artery disease less than males^3^. (ii) apparent age-related gender differences in ischemic heart disease mortality in England, Wales and the United States being attributed to a deceleration of associated events in older males, rather than an acceleration in post-menopausal women^32^. (iii) in a large, cross-sectional, population-based cohort study, independent associations of age and menopausal transition being associated with cardiovascular risk factors^31^. These scenarios do not fit seamlessly with a notion that the menopause solely imparts enhanced risks to female cardiovascular health.

Taking our *in vitro* experimental data in context with the above clinical studies, it is reasonable to propose that chronological aging is a prominent factor in influencing the altered vasodilatory effectiveness of estrogenic compounds. However, since the numbers recruited to the human study were small, we were not able to fully dissociate the effects of the menopause and aging on the altered effects of estrogenic compounds. Therefore, there is merit in performing a study, with larger numbers of human participants to explore further the relationship of age-related vascular dysfunction and altered estrogenic responsiveness. This would require longer than the duration of the current study, and/or the co-operation of multiple sites. Access to other sources of resistance vasculature, such as from gluteal biopsies, would extend the interpretations to another human vascular bed and enable comparisons with previous mixed gender studies focussing on essential hypertension^48,49^. Such studies will be beneficial in determining the feasibility of developing new regimens of estrogenic agents to treat vascular disease in post-menopausal women.

## Experimental procedures

### Animals

Mouse strains from the C57BL background (C57BL/Ircfa) were housed in the Comparative Biology Centre, Newcastle University, UK. All animal experimentation was performed under UK Home Office License (PIL 60/13278) and complied with the United Kingdom Animals (Scientific Procedures) Act 1986. Female mice from three separate age groups (3-, 24-, and 29-month-old) were utilized in the study. Mice were anaesthetized in an induction chamber using a rising concentration of 3% isoflurane/3 L oxygen per min. Once anesthesia was achieved the animal was culled by cervical dislocation and death confirmed as required by Animals (Scientific Procedures) Act 1986. The estrous stage of the mice was determined by vaginal smear^70^. Vaginal smears from aged mice were characterized by persistent di-estrous as described previously^71^. Small uterine and tail arteries were obtained from mice immediately after culling and placed in ice-cold tissue collection buffer (TCB) (in mM: 154 NaCl, 5.4 KCl, 1.2 MgSO_4_, 10 MOPS, 5.5 glucose, 1.6 CaCl_2_; pH 7.4). The experimental protocol was approved by Newcastle University Animal Welfare Ethical Review Body.

### Human subjects

Ethical approval was obtained from Newcastle and North Tyneside Research Ethics Committee 1 (10/H0906/71) to perform research on samples collected as part of the Uteroplacental Tissue Bank. Women who accepted to take part in the study gave written informed consent in compliance with the Helsinki Declaration and all experiments were performed in accordance with relevant guidelines and regulations. Patient characteristics are detailed in Table 2. In order to be considered postmenopausal, women had to report in the questionnaire that they had entered menopause in addition to reporting an amenorrhea of at least a year, which makes large-scale misclassification in this category less likely. Uterine arteries (identified on the myometrial surface under a dissecting stereomicroscope, at 1.5x magnification, Leica Microsystems, UK) from pre- and post-menopausal women were obtained from lower myometrial biopsies (~1 cm^3^) taken at the time of total hysterectomy, and placed directly into ice-cold tissue TCB. All experiments involving human samples were carried out in accordance with the relevant guidelines and regulations.

**Table 2:** Patient details.

Characteristics	Pre-MW (n = 26)	Post-MW (n = 16)
**Age** (**years**)	42.3 ± 0.5 (33–50)	61.9 ± 0.9 (50–75)*
**BMI** (**kg/m** ^**2**^)	28.3 ± 0.5	27.1 ± 0.4
**Smokers**	6 (30%)	2 (17%)
**Gravidity**	2.2 ± 0.2	2.5 ± 0.2
**Parity**	2.1 ± 0.1	2.0 ± 0.1
**Last menstrual period** (**years since**)	—	11.4 ± 0.8
**Ethnicity:**		
White	24	16
Not stated	2	
**Indication for surgery:**		
Menorrhagia	13	1
Prolapse	3	12
Abdominal pain	1	
Cystocele	2	1
Fibroids	3	
Prophylactic cancer reducing		1
Pelvic pain	2	
Urinary symptoms	1	
Stress incontinence	1	
Right iliac fossa pain		1
**Uterine Artery Resting diameter** (**μm**)**:**	303.2 ± 7.3	264.7 ± 10.4*

All values, apart from ethnicity and indication, are presented as mean ± SEM. Pre-MW = pre-menopausal women. Post-MW = Post-menopausal women. *P < 0.05 from pre-menopausal women (unpaired *t-test*).

### Isometric force measurements

Arteries were cut into 2–4 mm segments and mounted on a small vessel wire myograph (610 M; Danish Myotechnologies, Denmark) by inserting two tungsten wires (25 μm for mouse arteries and 40 μm for human arteries) through the lumen of the vessel. The first wire was attached to a micrometer (allowing adjustment of the lumen diameter during the normalization process) and the second wire was attached to a force transducer (which recorded changes in vessel wall tension). Vessels were normalized to a passive diameter equivalent to 0.9 of L_13.3_ kPa, as described previously^72–74^, and equilibrated in physiological salt solution (PSS) (in mM: 127 NaCl, 4.7 KCl, 2.4 MgSO_4_, 25 NaHCO_3_, 1.18 KH_2_PO_4_, 0.07 EDTA, 1.6 CaCl_2_, 6.05 glucose bubbled with 95% air/5% CO_2_; pH 7.4) at 37 °C for at least 20 minutes.

Vessels were pre-constricted with the thromboxane agonist 9,11-dideoxy-9a,11a- methanoepoxy prostaglandin F_2α_ (U4, Merck Millipore, UK) and allowed to reach a steady-state contraction. To assess endothelial function, human and mouse arteries were exposed to the endothelium-dependent vasodilators, bradykinin and acetylcholine, respectively. Arteries were then washed 3 times with PSS, pre-constricted with U4 (10^−6^M), and exposed to increasing doses (5 min duration each, of 10^−8^, 10^−7^, 10^−6.5^, 10^−6^, 10^−5.7^, 10^−5.3^, 10^−5^, 10^−4.5^M) of either 1,3,5-Estratriene-3, 17β-diol (17β-estradiol, non-specific ER agonist) (Sigma-Aldrich, USA), 4,4′,4″-(4-Propyl-[1 H]-pyrazole-1,3,5-triyl)-trisphenol (PPT, ERα-specific agonist), 2,3-bis(4-Hydroxyphenyl)-propionitrile (DPN, ERβ-specific agonist), (±)-1-[(3aR*, 4S*, 9bS*)-4-(6-Bromo-1,3-benzodioxol-5-yl)-3a,4,5,9b-tetrahydro-3*H*-cyclopenta[*c*]quinolin-8-yl]-ethanone (G1, GPER-specific agonist) (Tocris Biosciences, UK) or vehicle control (ethanol). This concentration range has been used in previous studies^9,10,12,41,75^. In separate experiments, the endothelium of mouse uterine arteries was rendered dysfunctional by gently abrading the intimal surface with human hair. Endothelial functional integrity, determined by the relaxant effect of acetylcholine (10^−5^M) on pre-constricted arteries, was 62.4 ± 4.2% for intact and 6.4 ± 1.0% for abraded arteries indicating that abrasion had evoked endothelial vasodilatory dysfunction. Cumulative concentration response curves were then elicited with 17β, PPT, DPN and vehicle control.

Data recorded (Myodaq; Danish Myotechnologies, Denmark) as active wall tension (Δ*T* in mN/mm) was transformed to active effective pressure (Δ*T*/(diameter/2000)) denoted by kPa.

### Passive structural characteristics

Murine arteries were mounted in a pressure myography system (Living Systems Instrumentation, USA) and allowed to equilibrate for 1 hour in calcium-free PSS (PSS with no added CaCl_2_ plus 1 mM EGTA) at 60 mmHg. Intravascular pressure was then reduced to 5 mmHg and subsequently increased to 10 mmHg, 20 mmHg and then in 20 mmHg steps to 120 mmHg. Diameters were allowed to stabilize for 5 minutes before proceeding to the next pressure step. Measurements of stable intraluminal diameter and left and right wall thickness (taken from 3 separate positions in the field of view, and averaged) were obtained at each pressure step.

Measurements of intraluminal diameter and wall thickness were used to calculate the following^76^:

- Area of the lumen (μm^2^): πr^2^ (where r is the radius)
- Area of the whole artery (μm^2^): π (lumen radius + one wall thickness)^2^
- Cross-sectional area of the vessel wall (μm^2^): vessel area − lumen area
- Stress (dyne/cm^2^): Pressure (1 mmHg = 1334 dyne/cm^2^) × radius/wall thickness
- Strain (ΔD/D_0_): Change in diameter from 5 mmHg/diameter at 5 mmHg
- Elastic modulus (E): E = β from the equation y = ae^βx^ used to fit an exponential trend-line to the stress plotted against strain.

### Western blotting

Arteries were cut into 5–10 mm segments and immediately snap frozen with 2-methylbutane (isopentane) cooled by liquid nitrogen and stored at −80 °C for further analysis of protein expression. Frozen samples were homogenized in cell lysis buffer (62.5 mM Tris – HCl, 2% SDS, 10% Sucrose) with 2% (vol/vol) protease inhibitor and 0.5% (vol/vol) phosphatase inhibitor at 5 µl per mg of tissue (minimum of 60 µl) and prepared for western blotting as previously described^77^. Thirty μg of protein was resolved by SDS-PAGE, transferred to a PVDF membrane for western blotting with ERα (Vector Labs #E-613 mouse monoclonal 1:200) or ERβ (Abcam #288 mouse monoclonal 1:500) and viewed chemiluminescently following incubation with HRP-goat anti-mouse-IgG (1:2000 Dakocytomation #PO447). Films were scanned (Umax powerlook scanner, Dallas, TX, USA) and bands were analyzed using the Intelligent Quantifier (Bio image systems, Jackson, MI, USA) software. Optical density (O.D.) of each band was measured relative to the O.D. of the positive control for each western blot. The background noise was subtracted from the O.D. of each band. PVDF membranes were stained with naphthol blue black in order to visualize actin expression and assess for equal protein loading between lanes.

### Statistics

All values were presented as mean ± SEM. Throughout the study, n refers to the numbers of women or mice. The passive structural characteristics of mouse arteries were compared using repeated measures two-way ANOVA (Fig. 1C–G). The parameters that showed significant differences with two-way ANOVA were then analyzed with a Bonferroni test for the comparison between age groups. The elastic modulus (E) was compared between murine age groups using an unpaired t-test (Fig. 1H). The vasodilatory effects of increasing concentrations of estrogenic compounds were compared using repeated measures two-way ANOVA. All maximum relaxations/contractions displayed in bar graphs were compared using an ordinary one-way ANOVA, with the exception of Fig. 4, in which an unpaired t-test was used to compare arteries from pre- and post-menopausal women. For the human studies, the trend between aging and the vasodilatory responses of estrogenic compounds was also examined using a scatter plot. A linear regression line was plotted and significant deviation from zero slope was determined by an *F-test*. Analysis was carried out using the GraphPad Prism (6.0) software (La Jolla, CA, USA). Significance was assumed at P < 0.05.

## Electronic supplementary material

Supplementary Figures
