# Supplementary material for: Estrogenic vascular effects are diminished by chronological aging
Source: Sci Rep. 2017 Sep 22;7:12153. doi: 10.1038/s41598-017-12153-5 (PMC5610317; doi:10.1038/s41598-017-12153-5)
Supplement: Supplementary file 1 — Supplementary Figures [file 41598_2017_12153_MOESM1_ESM.doc]

# **Estrogenic vascular effects are diminished by chronological aging**

Christopher J. Nicholson^1^, Michèle Sweeney^1^, Stephen C. Robson^1^ and Michael J. Taggart^1,2^

^1^Institute of Cellular Medicine, Newcastle University, Newcastle Upon Tyne, UK

^2^Cardiovascular Research Centre, Institute of Genetic Medicine, Newcastle University, Newcastle Upon Tyne, UK

**Corresponding author:** Christopher J. Nicholson PhD: Health Sciences Department, Boston University, 635 Commonwealth Avenue, Boston, MA 02215, USA. Telephone: 617-353-7567 Email: [cjnicho@bu.edu](mailto:cjnicho@bu.edu)

## Supplementary figures

**Supplementary Figure 1: Female uterine estrogenic relaxation does not involve GPER1.** Arteries were pre-constricted with U4 (10^-6^ M) and exposed to incremental doses (10^-8^ M – 10^-4.5^ M) of the GPER agonist G1 (Tocris, UK) (green half circles). The data presented previously in Fig 3 for 17β-estradiol (black diamonds) is included for reference. * P < 0.05 from vehicle control (VC) (two-way ANOVA).


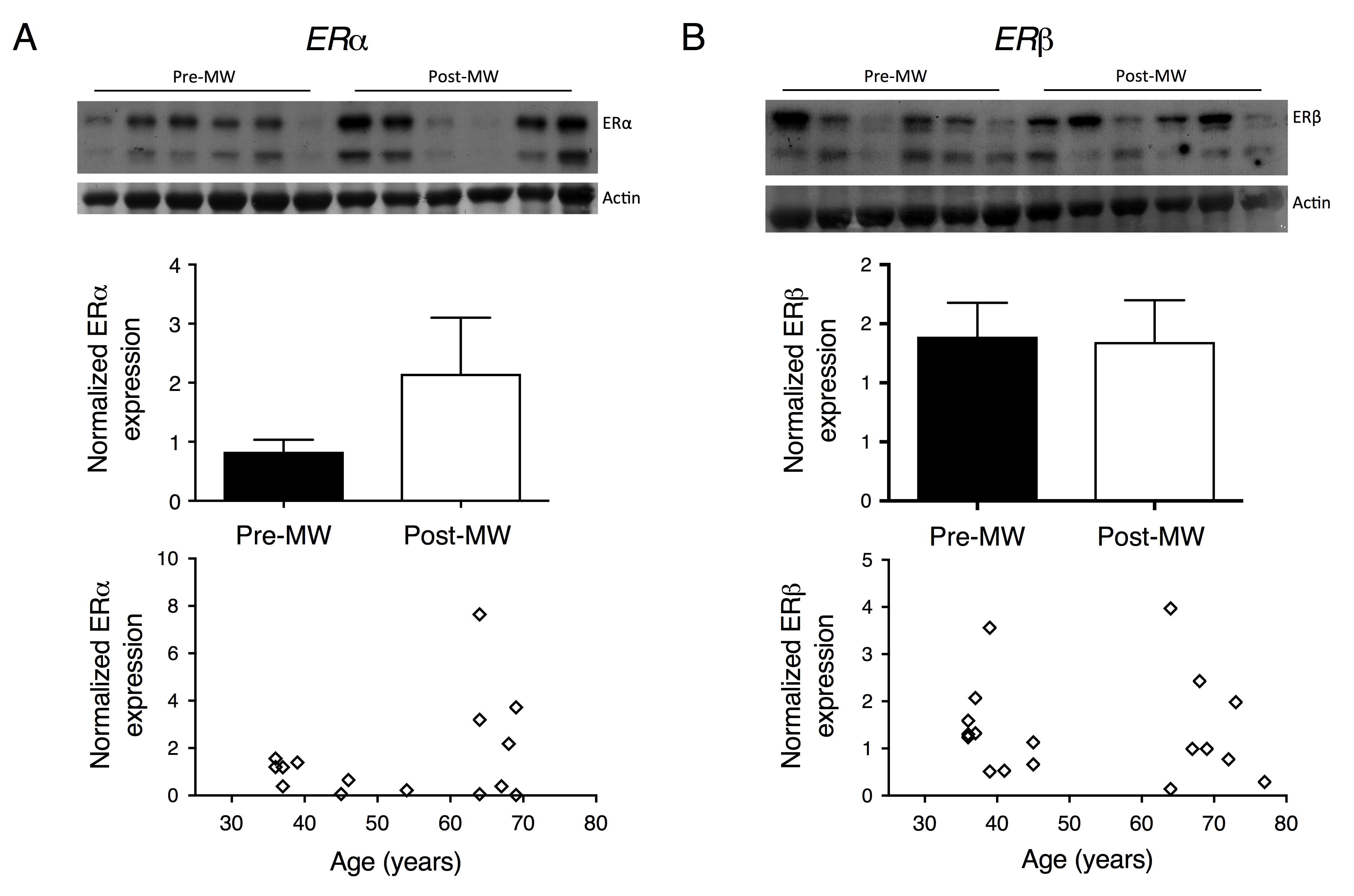


**Supplementary Figure 2: ER expression in myometrial arteries is unchanged after the menopause.** Typical western blots are displayed at the top (A & B). The expression levels (calculated as optical density of each band) of ERα (A) and ERβ (B) for each sample (n=8 and 12, respectively) were measured relative to the expression of the loading control (actin) and plotted against menopausal status (bar graph) or age (scatter plot). Please note that these blots were cropped (see Supplementary Figure 3 for full-length blots).

**Supplementary Figure 3: Full length blots cropped for representative figures.**
